# Plakin Expression in Serous Epithelial Ovarian Cancer Has the Potential to Impede Metastatic Spread and Epithelial–Mesenchymal Transition: A Comparative Expression Analysis of Immunohistochemical and In Silico Datasets

**DOI:** 10.3390/cancers16234087

**Published:** 2024-12-06

**Authors:** Tamsin Wesley, Ruth M. Escalona, George Kannourakis, Nuzhat Ahmed

**Affiliations:** 1 Fiona Elsey Cancer Research Institute, Ballarat, VIC 3353, Australia; tamsinwesley@gmail.com (T.W.); ruth@fecri.org.au (R.M.E.); george@fecri.org.au (G.K.); 2 Health Innovation and Transformation Centre, Mt Helen Campus, Federation University Australia, Ballarat, VIC 3353, Australia; 3 Centre for Reproductive Health, Hudson Institute of Medical Research, Clayton, VIC 3168, Australia; 4 Department of Molecular & Translational Science, Monash University, Clayton, VIC 3168, Australia; 5 Department of Obstetrics and Gynaecology, University of Melbourne, Parkville, VIC 3050, Australia; 6 Department of Surgery, St Vincent Hospital, University of Melbourne, Fitzroy, VIC 3065, Australia

**Keywords:** epithelial ovarian cancer, plakins, epithelial–mesenchymal transition, metastasis, ascites, in silico datasets

## Abstract

Epithelial ovarian cancer is aggressive and causes high mortality among women worldwide. Members of the plakin family are essential to maintain cytoskeletal integrity and key cellular processes. In this study we characterised the expression of plakins, particularly plectin (PLEC), periplakin (PPL), envoplakin (EVPL), and EMT-related proteins by immunohistochemistry in n = 48 patients’ samples to evaluate a potential correlation of plakin expression with EMT as EOC progresses. These tissue plakin and EMT expression analyses were further evaluated by in vitro cell line expression and correlated with the expression of these molecules using publicly available datasets such as Cancer Genome Atlas (TCGA) and Clinical Proteome Tumour Analysis Consortium (CPTAC) datasets. We demonstrate that the expression of PPL and PLEC plakins is decreased in high-grade compared to low-grade EOCs with mixed EMT marker protein expression. This is supported by the correlation of high PPL and PLEC expression with an epithelial rather than mesenchymal phenotype. Our data suggest a partial loss of plakin expression as EOC tumours progress. This may impact the connections of plakins with membrane-bound receptors, which impede the downstream signalling required for the initiation of EMT as the tumours progress.

## Simple Summary

Approximately 60% of patients diagnosed with epithelial ovarian cancer (EOC) die within the first five years due to disease recurrence. Identification of cytoskeletal proteins that may be involved in the epithelial-to-mesenchymal transition (EMT) may guide the accurate treatment and follow-up of patients with advanced EOC. The aim of this study was to evaluate whether there is a correlation between the expression of these cytoskeletal proteins and EMT as EOC progresses using primary EOC biopsies. We suggest that the expression of specific cytoskeletal proteins (plakins) is enhanced in early stages/grades of EOC but diminished as EOC progresses. However, the EMT marker proteins have a mixed expression, suggesting that plakin expression in primary tumours could impact the clinical progression and survival of patients with EOC.

## 1. Introduction

Epithelial ovarian cancer (EOC), particularly high-grade serous ovarian cancer (HGSOC), is the most common and deadly of the ovarian cancers, affecting over 1000 Australian women each year [1,2] and over 300,000 globally [3]. Five-year survival rates from EOC are as low as 30%, which has not changed over the past 30 years [1,4]. The most challenging aspect of this disease is the lack of success of precision surgery and the successive chemotherapy treatment to get rid of residual tumour to prevent terminal intra-abdominal spread of cancer [4,5,6,7,8]. A lack of effective early detection methods or distinctive symptoms contribute to frequent diagnosis in an advanced stage (FIGO 3-4), where the disease has spread to the adjacent peritoneal and distant organs [1]. Frequently patients respond to initial platinum/taxane-based chemotherapy, but relapse due to chemoresistance in a short time frame is common [9]. From that perspective, it is important to understand the biology and mechanisms of EOC spread so better strategies are developed to decelerate the spread of the disease.

Based on the histopathological features, EOC is divided into four subtypes: serous, endometrioid, clear-cell, and mucinous carcinomas. However, based on the origin and genomic features, EOC can also be divided into two subtypes: Type I and Type II tumours, of which Type II tumours are aggressive and prognostically fatal and are high-grade serous tumours thought to be derived from the intraepithelial carcinoma in the Fallopian tube [10]. Type I tumours, on the other hand, are low-grade serous tumours thought to be derived from tumour lesions on the ovarian surface epithelium and Müllerian inclusions [11] and include the endometrioid, clear-cell, and mucinous cancers. Type I tumours are generally diagnosed early, present in the early stages, and proliferate at a slow rate and have a good prognosis rate. When both tumours are compared, the Type II tumours classified as high-grade serous tumours are mostly diagnosed at an advanced stage. They have a high rate of proliferation and a fast and aggressive progress rate and a high degree of chromosomal instability with p53 mutation being present in 95% of cases [12].

In 70–80% of EOC patients, the tumour metastasises throughout the peritoneal cavity via the transcoelomic route, rather than the haematogenous and/or lymphatic routes observed in other cancers [13]. Transcoelomic metastasis occurs where tumour cells are detached, sloughed, or shed from the ovarian surface epithelium (OSE) or the Fallopian tube, as single or clumped cells nourished by the peritoneal ascites [14,15]. The shear pressure of ascites enables tumour dissemination to the common sites of the omentum, parietal and visceral peritoneum, and further abdominal sites such as the colon, liver, and pancreas [16,17,18,19]. The metastatic EOC cells embed onto mesothelial linings of the peritoneum [20,21], resulting in inflammatory cytokine production, enriching the tumour microenvironment (TME), enhancing tumour cell proliferation, and inhibiting innate and adaptive immune cell functions [22].

Whatever the route of metastasis, in cancer cells, EMT can drive metastasis, chemoresistance, and resistance to anoikis [23,24,25]. The transition is often identified as cells having impaired E-cadherin (ECAD) expression, as well as diminished expression of epithelial cell-associated molecule (EpCAM) and cell junction-associated proteins such as occludins, claudins, and integrin α6β4, with simultaneous increased expression of vimentin (VIM), N-cadherin (NCAD), fibronectin, and integrins β1 and β3 [26]. During the initiation or progression of EMT, epithelial cancer cells undergo early morphological, molecular, and functional changes resulting in the loss of epithelial cell–cell junctions and polarity and gain of a spindle-like mesenchymal morphology [27]. These functional changes facilitate cancer cell migration through stromal tissues and intravasation through blood vessels, leading to the dissemination of cancer cells to distant sites via hematogenous metastasis [28].

The EMT process has been associated with EOC initiation and progression [29,30]. It is thought that EMT drives the development of HGSOC precursors such as serous tubal intraepithelial carcinomas (STIC) and secretory cell outgrowths (SCOUT) [31,32,33]. However, cancer dissemination through transceolomic routes involves exfoliated cancer cells from ruptured lesions of the ovaries or the Fallopian tubes directly into the peritoneal cavity [13,34], which may not necessarily require the EMT process. However, once the cells have migrated and secondary metastatic lesions have been established, the process of EMT is reversed to mesenchymal–epithelial transition (MET) [35]. This continued transitional dynamics of EMT and MET contributes to the metastasis of EOC cells through the transcoelomic routes in the peritoneum [36]. Further to that, recent studies have shown that EOC cells can also exist in a hybrid EMT (E/M) state with characteristics of both epithelial and mesenchymal cells [36]. Cells in E/M state can readily transition into either epithelial or mesenchymal state depending on their functional needs [37], suggesting that both the processes of EMT/MET facilitate EOC progression.

Plakins are large cytolinker proteins that provide structural support to cells to maintain tissue integrity [38,39]. They join hemidesmosome and desmosome junction complexes to the plasma membrane, cytoskeleton, nucleus, and mitochondria of human cells [38]. Less studied is their role in signalling pathways that can regulate stress response, cellular growth, migration, invasion, and differentiation. In certain cancers, including pancreatic [40], bladder [41], colon [42], lung [43], and prostate [44], dysregulated expression of plakins is observed. The five core members of the plakin family are plectin (PLEC), desmoplakin (DSP), periplakin (PPL), envoplakin (EVPL), and epiplakin (EPPK1). We have recently shown the expression of PLEC, PPL, and EVPL in benign and malignant ovarian tumours [45]. As plakin family members such as PLEC, PPL and EVPL are central in maintaining cellular integrity by binding to the cytoskeleton and extracellular matrix, it is vital to understand the role of these molecules with cellular changes that accompany the EMT process. In addition, the expression of these plakins and their associated molecules are still ambiguous in ovarian tumours at different stages of disease progression.

This paper focuses on the expression of PLEC, PPL, EVPL and the classical EMT markers in different stages, grades, and types of serous epithelial ovarian tumours. We also analysed the expression profile of plakins in borderline serous tumours, described as typical proliferative tumours, and seen as precursors of World Health Organisation (WHO) Type I (low-grade serous tumours) [46,47]. In this study, we investigated the expression of plakins (PPL, EVPL, PLEC) in different stages, grades, and Types of serous ovarian tumours compared to benign tumours of the same origin. We attempted to understand the correlative relationship between the expression of plakins (PPL, PLEC, and EVPL) and the process of EMT by using immunohistochemistry and mRNA analysis on ovarian tumours, ovarian cancer cell lines, and in silico analyses.

## 2. Materials and Methods

### 2.1. Patient Tissue Collection, Treatment, and Ethics

The 37 human tissue samples used in this project are from surgical specimens donated with consent from the patients, diagnosed with EOC or benign tumours, under the Human Ethics application approved by The Research Ethics Committee of Royal Women’s Hospital (RWH#09/09), Melbourne, Australia. Each sample was obtained during initial tumour de-bulking surgery between 2000 and 2014 and was chemonaive. Twelve additional samples, including omentum, were acquired from the Victorian Cancer Biobank (VCB), Melbourne, Australia, under approval number VCB-17018. This project was approved for the Fiona Elsey Cancer Research Tissue Bank by Ballarat Health Services (Project ID: 37521) for studies involving humans.

Only epithelial serous ovarian tumours were obtained from patients diagnosed with ovarian cancer. Benign serous tumours were acquired from women undertaking abdominal hysterectomy or bilateral salpingo-oophorectomy due to previous medical conditions. Tissues were fixed in 4% paraformaldehyde at the time of collection. Patient information, such as tumour grade and stage of tumours, obtained from de-identified pathology reports. If WHO typing was not provided on histological reports, the equivalent WHO Type was assigned using the classification system provided by the World Health Organisation [46,47]. The histological reports provided the tumour description, FIGO stage, Silverberg–Shimizu grading, and p53 staining results. The p53 staining result from the histological report was also compared to in-house tissue staining to ensure consistency. Any case not allocated a confident WHO type was limited to analysis by Silverberg–Shimizu grade and FIGO stage.

### 2.2. Haematoxylin and Eosin (H & E) Staining of Tissues

This method was performed to determine the morphological features of tissue sections. Tissue sections, 3–4 µm thick, were heat-fixed onto glass slides (37 °C for 1 h) and de-waxed for 2–3 min of incubation in xylene × 2, 100% ethanol × 2, 80% ethanol × 2 and then 70% ethanol. Sections were stained with haematoxylin for 3–5 min. Sections were then dipped in acid-alcohol (1% HCl in 100% ethanol) for up to 10 s. The haematoxylin staining was blued in Scott’s tap water for 1 min, and the slide was counterstained with eosin for 30–60 s. The reverse dewaxing protocol was undertaken to dehydrate and clear the section and then xylene-soluble mountant was used to attach the coverslip.

### 2.3. Immunohistochemistry Staining of Solid Tissue Samples

A 3–4 μm section was collected on a DAKO Flex immunohistochemistry (IHC) slide, with heat fixation at 37 °C for one hour. The slides were dewaxed and rehydrated in a Ventana Benchmark Ultra automated IHC stainer (Oro Valley, AZ, USA). The staining process included antigen retrieval at pH 8.5, blocking of endogenous peroxidase activity, and incubating the tissue in primary antibody for 60 min. Each primary mouse anti-human antibody against PPL (G-1:sc-365530), EVPL (F-4:sc-137033), and PLEC (10F6:sc-33649) was used at a 1:100 dilution, was obtained from Santa Cruz Biotechnology (Dallas, TX, USA). This was followed by the Optiview HQ universal linker amplification step, treatment with horseradish peroxidase, and detection by DAB (3,3′-Diaminobenzidine). The slides were counterstained using Gill’s number 2 haematoxylin.

### 2.4. IHC Analysis

All slides for IHC analysis were scanned on a slide scanner suitable for use by ImageScope Aperio 12.3 software for analysis. The Aperio output ‘Positivity’ was used for quantification and controlled for background staining. The Aperio ‘Positivity’ measurement is calculated as a fraction of positively stained pixels to total pixels present, which is an equivalent measure of average intensity, which corrects for the size of the hand-drawn regions of interest. A minimum of three regions of interest (ROIs) were hand-drawn for each representative area of tumour cells for quantification. Three regions of interest were also drawn for the non-tumour stromal cells to establish the background staining for each section. An average of tumour cell positivity minus background staining was entered into GraphPad Prism 10.2.0 software for statistical analysis. The resulting data was treated as parametric (from a normal distribution), and thus a *t*-test or one-way ANOVA was applied depending on the number of groups compared. Where matched tissue data were available, the paired *t*-test or Pearson correlation test was used.

**Figure 1:**
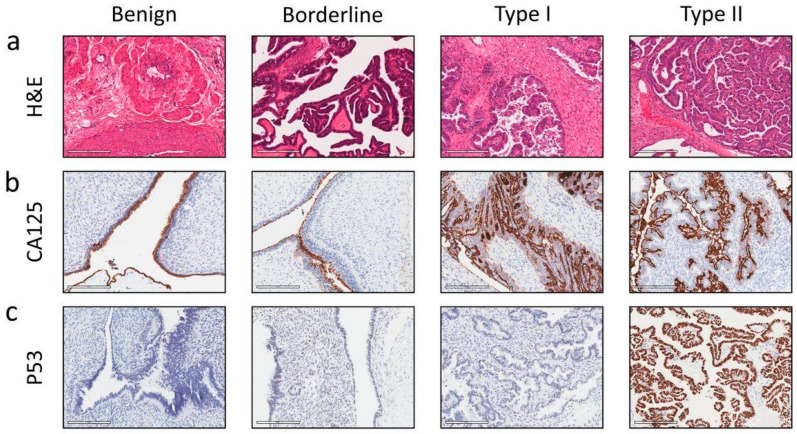
Representative images of benign and cancerous solid ovarian tumours evaluated by immunohistochemistry. Representative images of benign ovarian tissue, Silverberg borderline, WHO Type I and Type II tumours. (**a**) Cell morphology, H&E, (**b**) CA125 glycoprotein staining and (**c**) p53 immunostaining. Protein expression was deduced using immunohistochemistry, images were taken using a Leica DLMB microscope with attached Leica DFC450C camera and Leica Application Suite software (LAS, version 4.8.0), ×40 magnification, or from Aperio Imagescope software 12.8.

The tumour cells were identified for quantification based on the morphology, CA125, and p53 expression shown in Figure 1. To identify tumour cells within a tissue section, a haematoxylin and eosin stain (H&E) was used, which can show the following features of tumour cells: large nuclei (dark purple/blue), often multinucleated with distinct nucleoli and a low proportion of cytoplasm, and disordered size, shape, and growth in comparison to normal tissues (Figure 1a). As most ovarian tumour cells express high levels of CA125 glycoprotein, positive staining for CA125 by IHC, seen as a brown DAB stain in the cell membrane, enabled ovarian tumour cells to be identified within the tissue sections (Figure 1b). The cells can further be identified as tumour cells by abnormal p53 expression in cell nuclei, either as overexpression, with visible DAB stain (Figure 1c), common in EOC, or in limited cases, no staining, as some p53 mutations do not result in overexpression. When used together, the EOC cells present in a tissue section were identified and isolated on scanned images as a ‘region of interest (ROI)’ in analysis software.

The ROIs were kept consistent for each section and ensured that they contained cells representative of all tumour cells in the section. A ‘Positivity’ measurement was obtained for each case as the mean of three tumour-positive ROIs corrected for background (non-tumour) staining. For the VIM protein, the secondary-only control (IHC staining protocol, minus the primary anti-VIM antibody, was used for background correction as the non-tumour staining in VIM sections was universally high.

### 2.5. Ovarian Cancer Cell Lines

Established human OC cell lines: HEY [Cellosaurus HEY (CVCL_0297), commercial availability: https://www.cedarlanelabs.com/Cellutions], OVCAR4 [Cellosaurus cell line OVCAR-4 (CVCL_1627), commercial availability: https://www.merckmillipore.com], OVCAR5 [Cellosaurus OVCAR-5 (CVCL_1628), commercial availability: https://www.merckmillipore.com] and CAOV3 [Cellosaurus cell line Caov-3 (CVCL_0201), commercial availability: https://www.atcc.org/products/htb-75] were obtained from the laboratory of Professor David Bowtell, Peter MacCallum Cancer Centre, Melbourne, Australia. All cell lines were grown in either RPMI-1640 (Sigma-Aldrich, Sydney, Australia) or DMEM: MCDB medium (1:1) (Sigma-Aldrich, Sydney, Australia). Each growth medium was supplemented with 2 mM L-glutamine, 1% (*v*/*v*) streptomycin and penicillin, and 10% (*v*/*v*) foetal bovine serum (FBS) (Cell Sera, NSW, Australia). Cells were maintained at 37 °C in 5% CO_2_ humidity and were passaged at least twice a week once they reached a confluence of 65–80%.

### 2.6. mRNA Analysis

RNA was isolated using the RNasy ^®^Plus Mini Kit (Qiagen, Hilden, Germany) or the TaqMan ^®^Gene Expression Cells-to-CT™ Kit (Applied Biosystems, Mulgrave, Vic, Australia), according to the manufacturer’s instructions. RNA quality and concentration were analyzed using a NanoDrop 1000 spectrophotometer (Thermo Fisher Scientific, Melbourne, Australia). The RT2 First Strand Kit (Qiagen Pty Ltd., Melbourne, Australia) was used to remove any contaminating genomic DNA and synthesize cDNA with 1 μg RNA as per the manufacturer’s instructions. qPCR was performed using RT2 SYBR green master mix (Qiagen, Pty Ltd., Melbourne, Australia) run on a Rotor-Gene Q real-time cycler (Qiagen, Pty Ltd., Melbourne, Australia). Each 20 μL reaction mix contained 10 μL of RT2 SYBR green master mix, 0.8 μL of each of the forward and reverse primers (10 μM), 1 μL of cDNA, and 7.4 μL of RNase-free H_2_O. qPCR parameters were as follows: 95 °C for 10 min, 45 cycles of 95 °C for 15 s, and primer-specific annealing temperature for 30 s. Primer sequences are listed in Table 1. Samples were run three times in triplicate.

### 2.7. Protein Expression of PPL, PLEC, ECAD, NCAD and VIM Across Normal and Ovarian Tumours Using CPTAC Dataset

The CPTAC dataset was accessed via the UALCAN portal [48] that can perform in-depth analyses of proteins associated with different cancers. We used the CPTAC database (Proteomics, total protein) to analyse the protein expression profiles of selected proteins in the ovarian tumours and normal ovaries based on tumour stages and grades of the patients. The proteins analysed were PPL, PLEC, ECAD, NCAD, and VIM. The UALCAN website analysis measured statistical significance based on Student’s *t*-test with consideration of unequal variance [48].

### 2.8. Kaplan–Meier Curves, mRNA and Protein Correlation Accessed via TCGA Dataset

TCGA is a web-based podium that contains high-throughput genomic and proteomic data used to study prognostic associations of genes in different kinds of cancers, including EOC [49]. In the current study, this website was used to achieve survival analysis (Kaplan–Meier curves) of the above genes as a prognostic signature in Type II EOC [50]. Additionally, Pearson r correlation comparisons of mRNA and protein expression for plakins and an expanded set of EMT markers within Type II EOC were also produced.

## 3. Results

### 3.1. Plakin Protein Expression in Primary Untreated Ovarian Tumours by Immunohistochemistry

**Figure 2:**
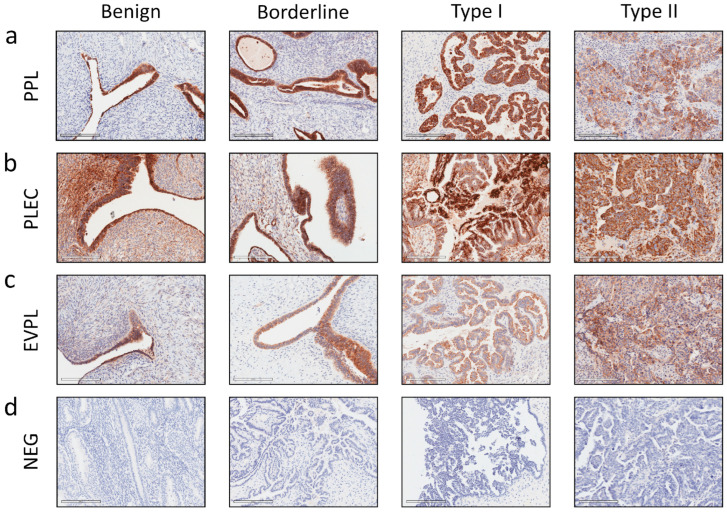
Representative images of plakin expression in benign and cancerous solid ovarian tumours evaluated by immunohistochemistry. Representative images of protein expression using immunohistochemistry in benign ovarian tissue, borderline, Type I and Type II tumours. (**a**) PPL, (**b**) PLEC, (**c**) EVPL immunostaining, and (**d**) IHC negative control image using secondary-only anti-mouse HRP-labelled DAB staining. Images were taken using a Leica DLMB microscope with attached Leica DFC450C camera and LAS software 4.8.0, ×40 magnification (D ×20 magnification) or from Aperio Imagescope software 12.3.

**Figure 3:**
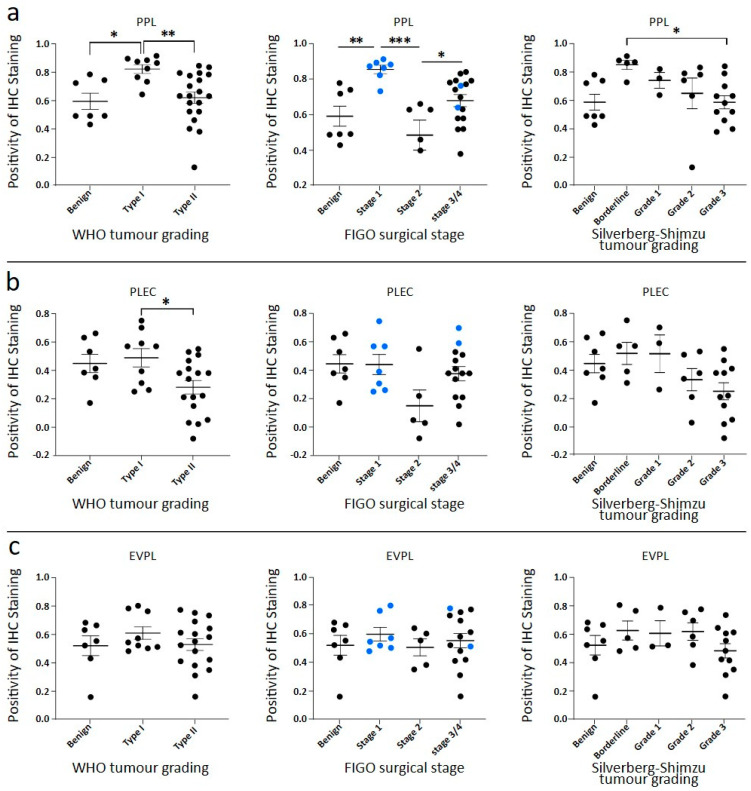
Quantitative analysis of plakins staining in benign ovarian tissues and serous ovarian tumours. Quantification of plakin protein expression staining using DAB immunohistochemistry. Positivity output from Aperio was normalized to background (non-epithelial cell) staining. Quantification of IHC staining: (**a**) PPL, (**b**) PLEC and (**c**) EVPL for each of cases sorted by WHO tumour Type, cases sorted by FIGO surgical stage, (blue data points represent WHO Type I cases) and cases sorted by Silverberg–Shimizu tumour grade. For PPL expression at a), statistical significance measured by one-way ANOVA, * *p* < 0.05, ** *p* < 0.01, *** *p* < 0.005. For PLEC at (**b**), statistical significance measured by one-way ANOVA, * *p* < 0.05. For EVPL at (**c**), statistical significance was measured by one-way ANOVA; no significance was determined between the groups.

The staining of primary tumours showed a range of plakin expression levels in benign ovarian tissues and epithelial ovarian tumours of different stages (FIGO), grades (Silverberg–Shimizu), and Types (WHO classification of serous ovarian tumours) (Figure 2 and Figure 3). PPL protein expression in these tissues is shown in Figure 2a and Figure 3a. In Figure 2a, the PPL staining of the normal layer of epithelial cells in the benign tissue sample is strong, with no positive stain seen in the stroma. The staining is present in the cytoplasm and cell membrane of epithelial cells. Strong staining is seen in borderline and Type I tumour cells, whereas the staining in Type II tumour cells is more diffuse and less intense.

Overall, in our samples, enhanced expression of PPL was noted in type I tumours compared to benign controls (Figure 3a). The Type II cases had lower PPL expression than Type I, with a similar mean expression to benign controls (Figure 3a). The change in PPL expression was significant between the Type I (low grade) and Type II (high grade) tumours (Figure 2a and Figure 3a), which may be consistent with the dissimilar genetic profiles suggested for these classifications [10,11]. When separated by FIGO surgical stage (Figure 3a), most of the Type I cases fell into stage 1 (confined to ovaries/uterus) and the Type II mostly fell into stages 3 and 4 (demonstrated metastases) (Figure 3a). Subsequently, a similar difference in PPL expression was observed between benign and Type I and benign and stage 1 tumours (Figure 3a).

When cases were stratified according to Silverberg–Shimizu grading (Figure 3a), increased expression of PPL in borderline tumours compared to benign controls was seen (Figure 3a), with a subtle trend of reducing PPL with increasing grade present. Consequently, PPL expression in grade 3 was similar to benign controls but significantly different from borderline (Figure 3a). An example of PPL expression in a Silverberg borderline case is shown in Figure 2a.

PLEC staining is shown in Figure 2b and analysis in Figure 3b. In Figure 2b, the benign tissue has strong staining in the cell membrane of epithelial cells, with some cells in a transverse orientation at the top of the field shown. Some background staining of non-epithelial cells can be seen. The Type I tumour cells in Figure 2b show strong membrane staining, continuing into the cytoplasm. Whereas Type II staining is more limited to the cell membrane, with the representative image showing high background staining. When undertaking analysis, the full range of staining patterns present in tumour cells was included in the regions of interest quantified. There was no increase in PLEC expression between benign and Type I, but a significant decrease in PLEC expression between Type I and Type II was observed (Figure 3b), which was similar to PPL expression (Figure 3a). When the cases were presented according to the surgical stage, the average PLEC expression across all cases remained steady, except for stage 2, with low PLEC expression (Figure 3b). When cases were ordered by Silverberg–Shimizu grade, it showed PLEC expression above benign levels for the borderline and grade 1 groups and below benign levels for grades 2 and 3 without significant differences (Figure 3b).

The pattern of cell staining observed with EVPL staining of benign cells in Figure 2c is diffuse across the epithelial cells, with no distinct membrane outline. Background staining is quite low in benign tissues (Figure 2c). The diffuse pattern was also seen with Type I tumour cells and a sporadic, sometimes reduced expression within Type II cells (Figure 2c). When our cases were compared by WHO Type, no significant differences were detected in the mean EVPL expression between benign, Type I, and Type II tumours (Figure 3c). The cases were presented by FIGO stage and by Silverberg–Shimizu grade in Figure 3c, with no strong trends or significant differences seen.

### 3.2. Expression of EMT Associated ECAD, NCAD and VIM in Primary Untreated Ovarian Tumours by Immunohistochemistry

**Figure 4:**
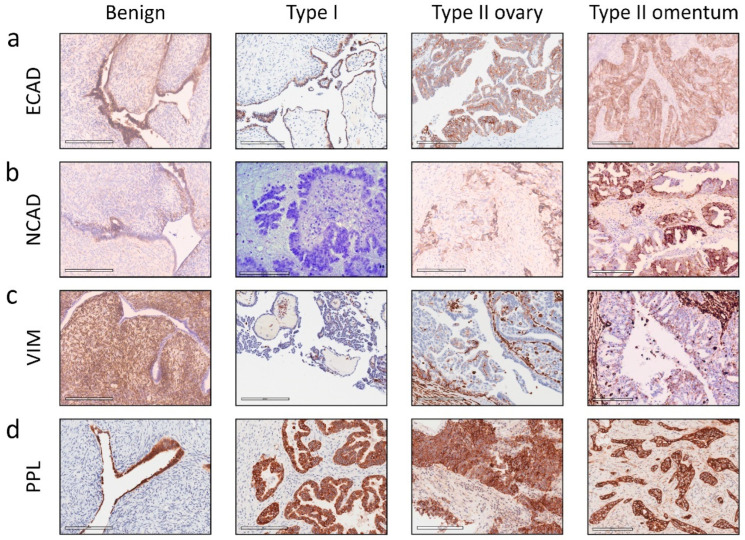
Representative images of benign and cancerous solid ovarian tumours evaluated by immunohistochemistry for EMT markers. Representative images of benign ovarian tissue, WHO Type I tumours and WHO Type II tumour deposits in both ovary and omentum (pre-chemotherapy). (**a**) ECAD, (**b**) NCAD, (**c**) VIM, and (**d**) PPL immunostaining. Protein expression staining using DAB immunohistochemistry, images taken from Aperio slide scans, ×20 magnification.

**Figure 5:**
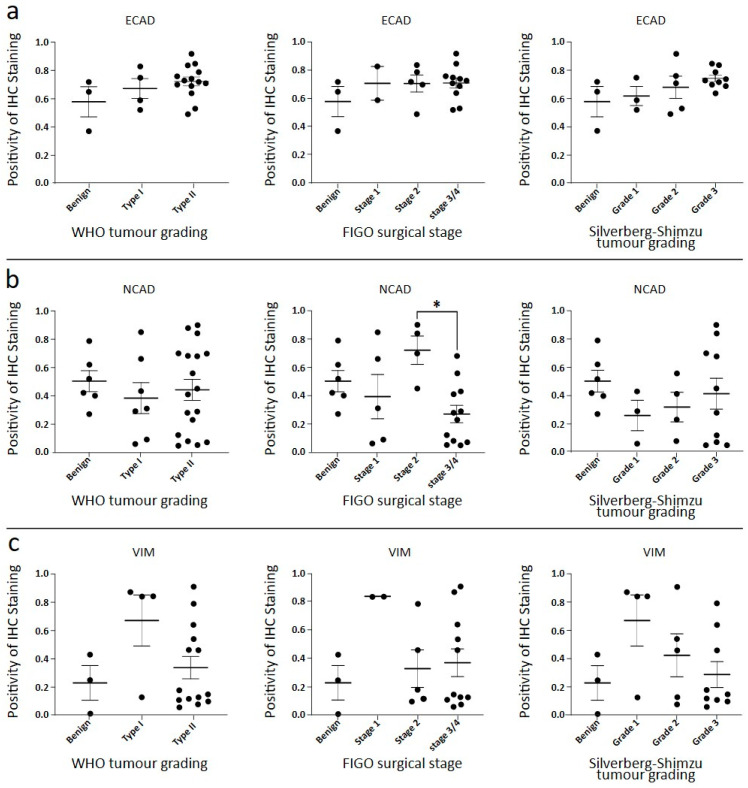
Quantitative analysis of EMT marker staining in benign tissues and serous ovarian tumours. EMT marker protein expression staining using DAB immunohistochemistry. Positivity output from Aperio was normalized to background staining. Quantification of IHC staining for (**a**) ECAD, (**b**) NCAD, and (**c**) VIM for cases sorted by WHO tumour grade, cases sorted by FIGO surgical stage, and cases sorted by Silverberg–Shimizu tumour grade. For ECAD at (**a**), statistical significance measured by one-way ANOVA, no significance found. For NCAD at (**b**), statistical significance measured by one-way ANOVA, * *p* = 0.015. For VIM at (**c**), statistical significance measured by one-way ANOVA; no significance was found.

To compare plakin expression with EMT, a subset of the primary tumour set was stained by IHC for ECAD, NCAD, and VIM (Figure 4). This figure also included metastasised tumour deposit samples from the omentum, also collected during debulking surgery. ECAD is limited to epithelial cells, and in Figure 4a, high ECAD expression is present in the cytoplasm and cell membrane, along with moderate background non-epithelial staining. In Figure 4a, the pattern of ECAD staining is very similar in Type I tumour cells compared to the benign sample. In the Type II tumour cells (Figure 4a), the staining pattern is not uniform, with differing levels of cytoplasmic staining and intermittent strong membrane staining. Across the samples, total ECAD expression appeared consistent, but with a trend towards increased expression in both Type II and grade 3 tumours (Figure 5a). When cases were stratified by surgical stage, all tumours appeared to have higher ECAD expression than the benign controls (Figure 5a). The Aperio software was not able to discriminate against staining by location, i.e., cell membrane, nucleus, or cytoplasm. This prevented additional analysis of the ratio of cytosolic versus membrane protein expression.

The benign tissue shows strong cytoplasmic expression of NCAD in the epithelial cells and some background staining in the non-epithelial regions (Figure 4b). The Type I cases have a wide range of NCAD expressions, with the image in Figure 4b an example of low NCAD expression across the sample. The Type II tumour cells have strong membrane staining of NCAD, with moderate diffuse staining in the non-epithelial cells. Overall, the mean expression of NCAD is unchanged between benign, Type I, and Type II tumours (Figure 5b). In FIGO stage groups, stage 1 is lower in NCAD expression than benign controls, and stage 2 is higher than benign tumour samples (Figure 5b). However, NCAD is reduced again in stages 3 and 4, significantly different from stage 2, shown in Figure 5b. The trend of NCAD expressions in Silverberg–Shimizu grades is similar to the WHO tumour Types, where benign tissue expression is higher than early grade/Type I tumours, then NCAD expression increases with disease grade, such that grade 3/Type II tumours have higher expression than grade 1/Type I tumours (Figure 5b). In short, a varied expression of NCAD was observed with no clear trend noted in malignant ovarian compared to benign tumours.

VIM is an intermediate filament (IF) protein that is not highly expressed in epithelial cells, but increased VIM indicates a cell’s transition towards a mesenchymal phenotype [23]. Additionally, the non-epithelial cells in our samples stained either moderately or strongly for VIM (Figure 4c). Subsequently, the identified normal epithelial cells in benign samples (Figure 4c) and the malignant epithelial cells in the tumour samples were controlled for background staining using the secondary-only controls demonstrated in Figure 2d. In Figure 4c, the benign epithelial cells are not stained with the VIM antibody. VIM staining is low in the Type I tumour clusters, identifiable by their strong haematoxylin-staining nuclei and low cytoplasmic content. The Type II tumour deposits in Figure 4c are less dense than Type I and have stromal infiltrates that stain positively for VIM. This supporting stroma was not included in the regions of interest analysed.

Overall, no significant differences in VIM expressions were seen when the samples were stratified by Type, stage, and grade as shown in Figure 5c. Very high staining of VIM in stromal cells is seen in Figure 4c, making it challenging to see the epithelial tumour cells. However, regions of interest were carefully chosen to reflect the highest concentration of tumour cells for analysis. The Type I cases had higher VIM expression than the benign controls, and a wide range of expression levels was observed in Type II cases. Two cases that presented with stage 2 tumour spread had a high level of VIM expression that contrasted with the overall consistent mean expression across all the other groups in Figure 5c. Similarly, a small number of high-VIM cases fell into the grade 1 Silverberg–Shimizu group, with a possible trend towards lower expression in grades 2 and 3, but the differences are not strong enough for significance. In short, a decreasing trend of VIM was noted in higher grades, stages, and Types compared with lower-rated tumours.

**Figure 6:**
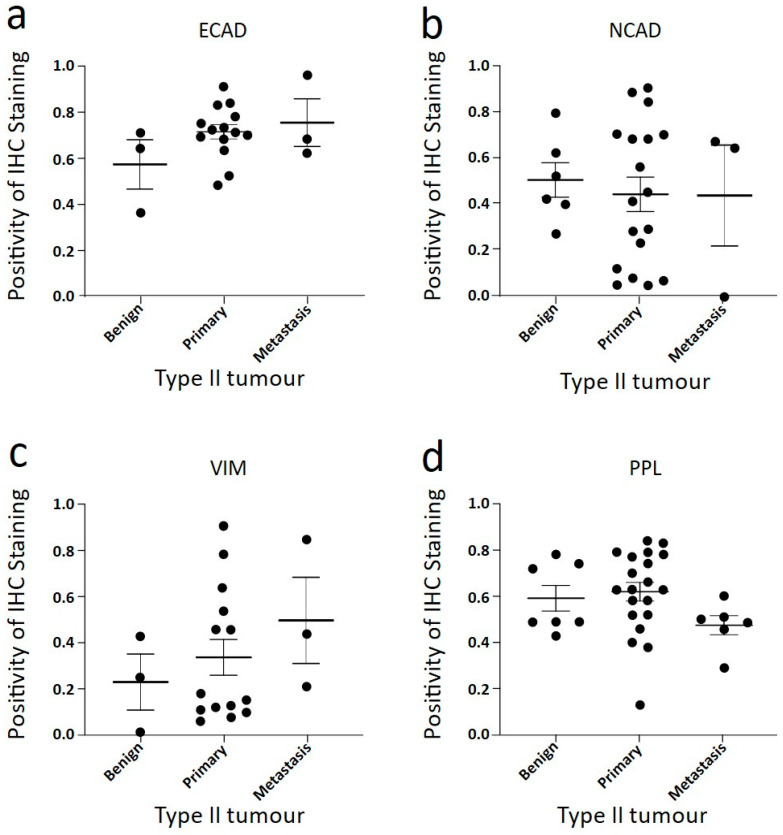
Quantitative analysis of EMT marker staining in benign tissues, ovarian (primary) WHO Type II cases and omental (metastatic) deposits. Analysis of EMT marker staining in benign tissues, ovarian (primary) WHO Type II cases and omental (metastatic) deposits. (**a**) ECAD stained WHO Type II cases sorted by origin of tumours, (**b**) NCAD stained WHO Type II cases sorted by origin of tumours, (**c**) VIM stained WHO Type II cases sorted by origin of tumours and (**d**) PPL stained WHO Type II cases sorted by origin of tumours. Statistical significance measured by one-way ANOVA; no significance was found.

We next compared the expressions of ECAD, NCAD, VIM, and PPL in benign, primary (ovary), and metastatic omentum tissues. Also shown in Figure 4a–c are representative images of ovarian tumour cells present in omental tissue, stained for EMT markers (n = 3–5). Figure 4d includes the PPL staining of Type II tumour cells in the omentum. Analysis of these proteins by tumour subtype is shown in Figure 5, and comparison between ovarian and omental Type II tumour deposits is demonstrated in Figure 6. The benign and Type I images in Figure 4d are the same as in Figure 2a.

ECAD and NCAD expression varied between benign, primary (ovary), and metastatic (omentum) deposits (Figure 4a,b). As the benign and primary tumours are the same as the benign and Type II presented in Figure 2 and Figure 4, their comparison is presented above. Both ECAD and NCAD expressions are very similar in metastatic tumour deposits compared to primary tumours. There may be increased VIM expression in metastatic tumour deposits in the omentum, possibly due to greater involvement of stromal cells supporting the tumour cell nests (Figure 4c and Figure 6c). In Figure 4d and Figure 6d, the metastasized cells showed slightly less PPL than those found at the primary site, suggesting less maturity of cell–cell junctions and possibly less dense cell connections due to the smaller number of cells in individual deposits.

**Figure 7:**
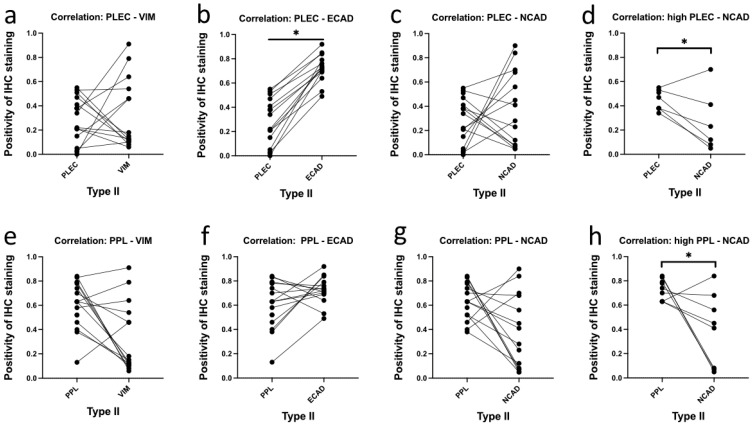
Correlation of PLEC and PPL with EMT markers in WHO Type II primary tumour samples. Pearson correlation analysis of PLEC and PPL staining with EMT marker expression in Type II tumours from IHC staining. Top row: (**a**) PLEC vs. VIM, (**b**) PLEC vs. ECAD * *p* < 0.05, (**c**) PLEC vs. NCAD, (**d**) high PLEC (cases above mean PLEC expression) vs. NCAD * *p* < 0.05. Bottom row: (**e**) PPL vs. VIM, (**f**) PPL vs. ECAD, (**g**) PPL vs. NCAD, (**h**) high PPL (cases above mean PPL expression) vs. NCAD * *p* < 0.05.

A correlation test between PLEC or PPL and the EMT marker expression quantified by IHC staining is shown in Figure 7. This was limited to our in-house WHO Type II cases with matched data measurements across the proteins of interest. This revealed a significant correlation between high PLEC and high ECAD expression (Figure 7b). Also, significant were two correlations with both high PLEC and high PPL with low NCAD expression (Figure 7d,h). A trend of high PLEC and low VIM expression was observed (Figure 7a), suggesting that PLEC is retained in cases where EMT changes are not yet evident. This trend was also seen when comparing PPL to VIM in Figure 7e.

### 3.3. Expression of PPL, EVPL and PLEC in Ovarian Cancer Cell Lines

**Figure 8:**
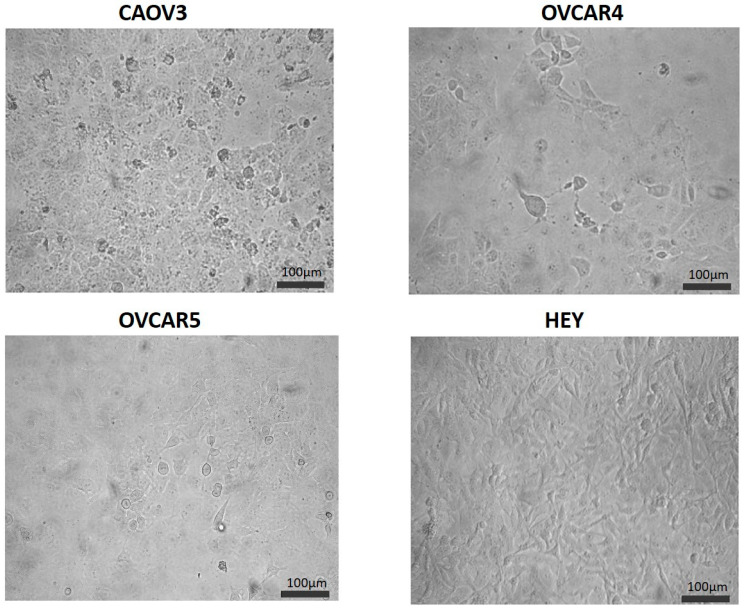
Morphological features of ovarian cancer cell lines. Representative phase contrast images of CAOV3, OVCAR4, OVCAR5, and HEY cell lines grown in monolayer culture. Magnification: 20× magnification, scale bar = 100 um.

To further enable comparison of plakin expression and its role in ovarian cancer progression, four different epithelial ovarian cancer cell lines were chosen, and an assessment of expression was undertaken at the mRNA level. The cell lines chosen for investigation included HEY—ovarian papillary cystadenocarcinoma origin (CVCL_0297), CAOV3—adenocarcinoma origin (CVCL_0201), OVCAR4 (CVCL_1627), and OVCAR5 (CVCL_1628), both high-grade ovarian adenocarcinoma, derived from ascites. The OVCAR4 cell line was established from a patient who had undergone chemotherapeutic treatment; OVCAR5 was from a different patient prior to their chemotherapy. Of these cell lines, morphologically COAV3 is a slow-growing homogenous epithelial cell line, while both OVCAR4 and OVCAR5 represent a mixture of epithelial and mesenchymal cells, and HEY is a homogenous population of mesenchymal cells (Figure 8).

**Figure 9:**
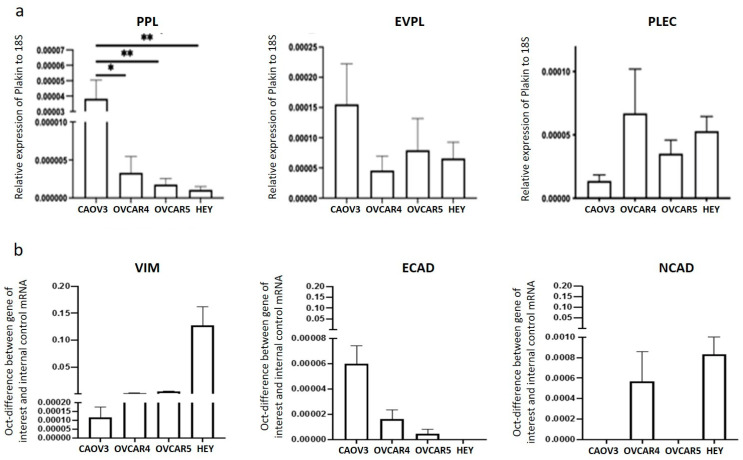
mRNA expression of plakins and EMT markers in ovarian cancer cell lines. (**a**) mRNA expression of PPL, EVPL, and PLEC in CAOV3, OVCAR4, OVCAR5, and HEY cell lines were measured as described in Methods. Data is expressed as mRNA expression of gene of interest fold-change relative to 18S rRNA housekeeping gene. n = 3, biological and technical triplicates were performed. One-way ANOVA performed, * *p* < 0.05, ** *p* < 0.01. (**b**) mRNA expression of VIM, ECAD, and NCAD was evaluated as described in the methods. Data are expressed as gene of interest fold-change relative to 18S rRNA housekeeping gene. n = 3, biological and technical triplicates were performed. Biological and technical triplicates were performed, n = 3, statistical analysis was not performed.

Plakin mRNA is not highly expressed in confluent monolayer cultures of OVCAR4, OVCAR5, CAOV3, and HEY cell lines (Figure 9a). The expression of PPL and EVPL was highest in CAOV3, an slow-growing epithelial cell line, and was lower in the remaining, faster-growing cell lines, OVCAR4, OVCAR5, and HEY. No significant differences were found in EVPL and PLEC mRNA expression amongst the cell lines (Figure 9a).

The mRNA expression of the classical EMT markers, ECAD, NCAD, and VIM, for comparison, is presented in Figure 9b. Our results support observations that the epithelial-like nature of CAOV3 cells had the highest ECAD and lowest mRNA expression for both NCAD and VIM (Figure 9b). This is consistent with the highest expression of PPL in this cell line (Figure 9a). In our observation, CAOV3 grows prohibitively slowly and produces extraordinarily little tumour growth in mice (unpublished data). OVCAR4 and OVCAR5 showed similar levels of VIM mRNA (Figure 9b), whereas OVCAR4 had higher levels of both ECAD and NCAD mRNA than OVCAR5 (Figure 9b). The HEY cell line had the highest VIM and NCAD mRNA detected (Figure 9b), with ECAD mRNA barely detected (Figure 9b). These observations are consistent with the mesenchymal morphology of this cell line demonstrated in Figure 8.

### 3.4. In Silico Analysis of PPL, PLEC, ECAD, NCAD, and VIM Protein Expression in Normal Ovaries and Different Stages and Grades of Ovarian Tumours Derived from CPTAC Samples

**Figure 10:**
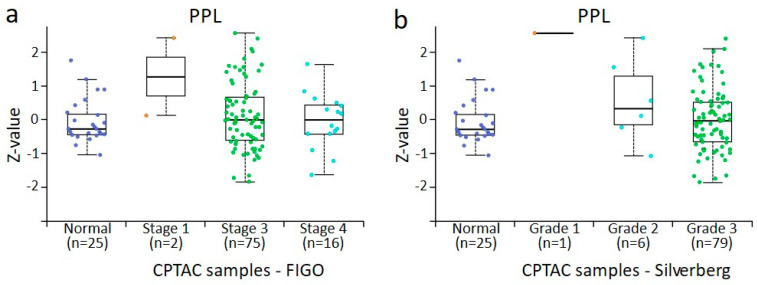
PPL protein expression from CPTAC dataset in ovarian cancer by surgical tumour stage (FIGO) and Silverberg grade. All cases are from CPTAC from the UALCAN [48]. (**a**) Ovarian tumours compared by surgical stage, (**b**) ovarian tumours compared by Silverberg grade. Insufficient data points were available for statistical comparison of all grades and stages. Student’s *t*-test with consideration of unequal variance with *p* < 0.001 was found when surgical stage 3 and stage 4 were compared and Silverberg grade 2 versus grade 3, where both grades commonly fall within the WHO Type II definition.

**Figure 11:**
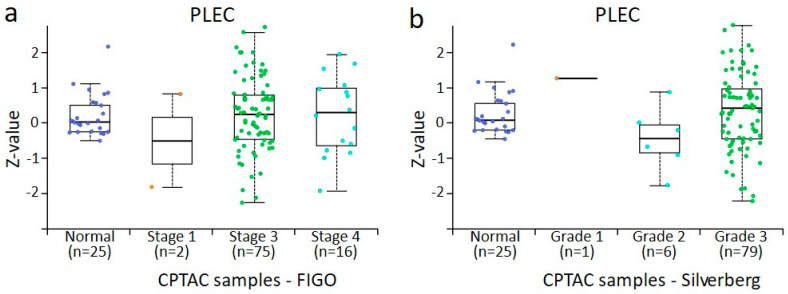
PLEC protein expression from CPTAC dataset in ovarian cancer by surgical tumour stage (FIGO) and Silverberg grade. All cases are from CPTAC from the UALCAN [48]. (**a**) Ovarian tumours compared by surgical stage, (**b**) ovarian tumours compared by Silverberg grade. Insufficient data points were available for statistical comparison of all grades and stages. Student’s *t*-test with consideration of unequal variance with *p* < 0.001 was found when surgical stage 3 and stage 4 were compared and Silverberg grade 2 versus grade 3, where both grades commonly fall within the WHO type II definition.

The data accrued from the in-house collected samples could not be directly compared with the data obtained from CPTAC samples because of the differences in the groups analysed. In the CPTAC data, accessed via the UALCAN website, 45 normal ovaries instead of benign ovarian tumours (used in-house accrued samples) were included for comparison with primary tumours. However, a similar trend of changes in the expression was noted. For plakins, only data on PPL and PLEC were available. In the case of PPL, a trend in increased PPL expression was noted with the stages and grades of tumours compared to normal ovaries, although it was only significant between stage 3 and normal ovaries, stage 3 and 4 tumours, and grades 2 and 3 tumours (Figure 10a,b). However, as there were only n = 2 tumours in stage I and n = 1 in grade 1 tumour groups, no significance could be determined between these groups. In Figure 10b, when grades 1 and 2 are pooled, it suggests that PPL expression in grades 1 or 2 is higher than in normal tissue and higher than in grade 3 cases. Seeing that Silverberg grade 3 often translates to WHO Type II, this is consistent with our observations in Figure 3a. In the case of PLEC, a significant increase between normal ovaries and stage 3 tumours and stage 3 and 4 tumours was observed (Figure 11a). In the case of grades, a significant increase in PLEC expression was noted in grade 3 compared to grade 2 tumours, with a trend of decreasing expression in grade 2 and increasing in grade 3 compared to normal ovaries (Figure 11b).

**Figure 12:**
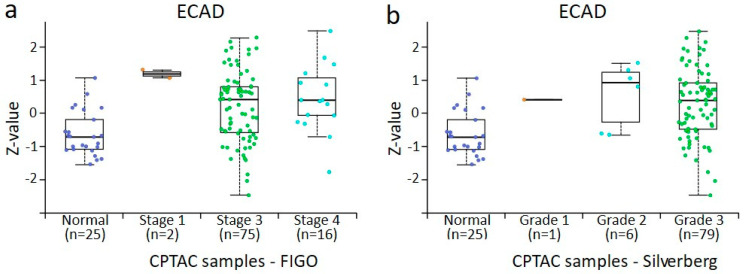
ECAD protein expression from CPTAC dataset in ovarian cancer by surgical tumour stage (FIGO) and Silverberg grade. All cases are from CPTAC from the UALCAN [48]. (**a**) Ovarian tumours compared by surgical stage, (**b**) ovarian tumours compared by Silverberg grade. Insufficient data points were available for statistical comparison of all grades and stages. Student’s *t*-test with consideration of unequal variance with *p* < 0.001 was found when normal tissue was compared to surgical stage 3 and stage 4, and between stage 3 and stage 4. In normal versus grade 2 *p* < 0.05, normal versus grade 3 *p* < 0.001, and Silverberg grade 2 versus grade 3 *p* < 0.001, where both grades commonly fall within the WHO Type II definition.

**Figure 13:**
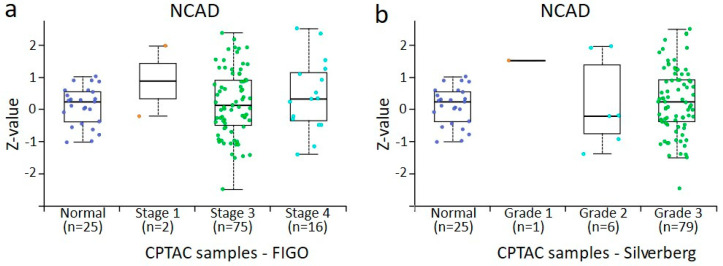
NCAD protein expression CPTAC dataset in ovarian cancer by surgical tumour stage (FIGO) and Silverberg grade. All cases are from CPTAC from the UALCAN [48]. (**a**) Ovarian tumours compared by surgical stage, (**b**) ovarian tumours compared by Silverberg grade. Insufficient data points were available for statistical comparison of all grades and stages. Student’s *t*-test with consideration of unequal variance with *p* < 0.001 was found when surgical stage 3 and stage 4 were compared and Silverberg grade 2 versus grade 3, where both grades commonly fall within the WHO Type II definition.

**Figure 14:**
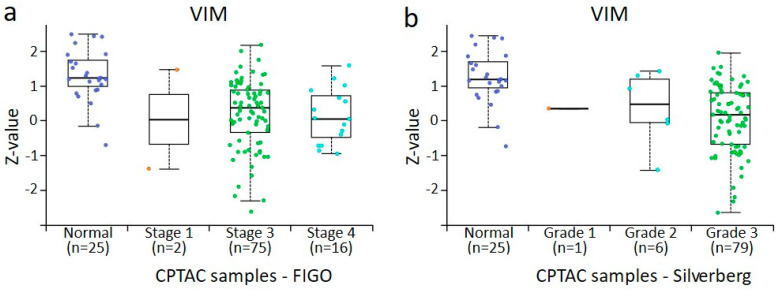
VIM protein expression from CPTAC dataset in ovarian cancer by surgical tumour stage (FIGO) and Silverberg grade. All cases are from CPTAC from the UALCAN [48]. (**a**) Ovarian tumours compared by surgical stage, (**b**) ovarian tumours compared by Silverberg grade. Insufficient data points were available for statistical comparison of all grades and stages. Student’s *t*-test with consideration of unequal variance with *p* < 0.001 was found when normal versus surgical stage 3, and stage 3 versus stage 4 were compared. In normal tissue versus Silverberg grade 3 *p* < 0.001, and Silverberg grade 2 versus grade 3 *p* < 0.001, where both grades commonly fall within the WHO Type II definition.

The expression profile of EMT-associated ECAD, NCAD, and VIM in CPTAC samples was consistent with what was observed in our in-house samples analysed by IHC (Figure 12, Figure 13 and Figure 14). There was a trend in the increase in expression of ECAD in stages 1, 2, and 4 tumours compared to normal ovaries in CPTAC samples, consistent with the trend observed between benign ovarian tumours and various stages and grades of tumours (Figure 12 and Figure 5a). A similar observation was noted in NCAD expression, where the expression of NCAD in different stages and grades of tumours showed varied expression compared to normal ovaries (Figure 13), consistent with IHC staining observed in in-house samples (Figure 5b). Contrary to that, the expression of VIM decreased in different stages and grades of ovarian tumours compared to normal ovaries (Figure 14a,b), compared to IHC data in our in-house samples, which showed an increasing trend in different stages and grades of tumours compared to benign ovaries (Figure 5c).

### 3.5. Association of Plakins with EMT Markers and Other Proteins: Extrapolation of Publicly Accessible Database Findings

**Figure 15:**
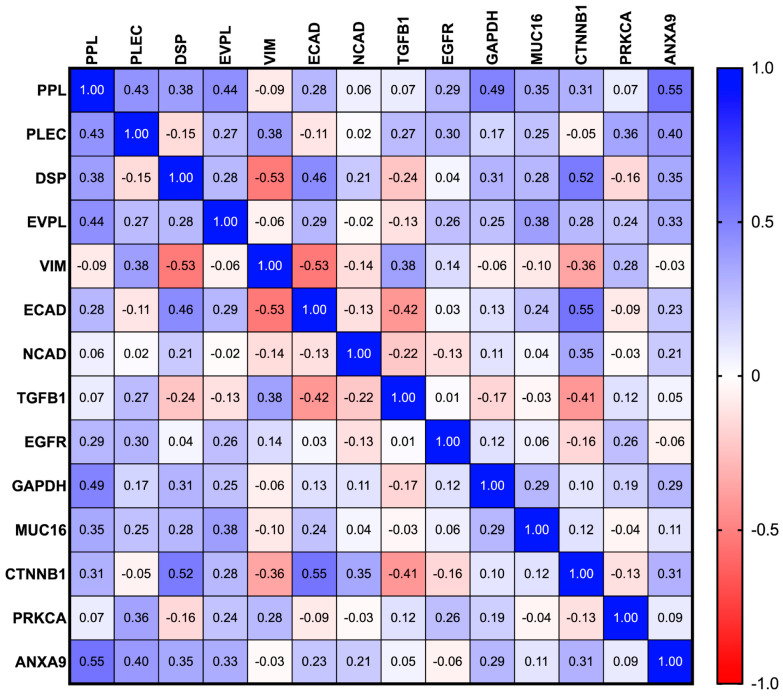
Pearson r correlation of the protein expression of plakins with EMT markers and plakin associated proteins from TCGA dataset. All cases are WHO Type II ovarian tumours with p53 mutations from the dataset titled Ovarian Serous Cystadenocarcinoma (TCGA, Nature 2011) [49]. Data analysis and correlation plot are produced with GraphPad Prism 10.2.0 software. Comparison of plakin proteins of interest and classical and alternative markers of EMT. Weak correlation between 0 and 0.3 (or −0.3), moderate between 0.3 and 0.5, and very strong above 0.5, n = 79.

**Figure 16:**
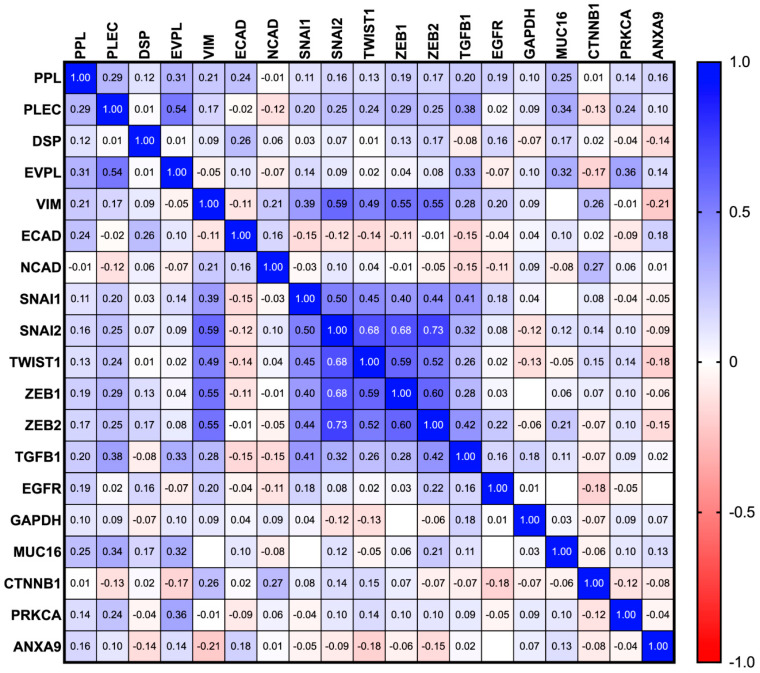
Pearson r correlation of the mRNA expression of plakins with EMT markers and plakin associated proteins from TCGA dataset. All cases are WHO Type II ovarian tumours with p53 mutations from the dataset titled Ovarian Serous Cystadenocarcinoma (TCGA, Nature 2011) [49]. Data analysis and correlation plot were produced with GraphPad Prism 10.2.0 software. Comparison of plakin mRNA of interest and classical and alternative markers of EMT. Weak correlation between 0 and 0.3 (or −0.3), moderate between 0.3 and 0.5, and very strong above 0.5, n = 240.

In this study, we interrogated the TCGA database to analyse the correlation of protein and mRNA expression of plakins with EMT-associated markers by using Pearson correlation analysis (Figure 15 and Figure 16). The analysis considered a greater number of plakins and EMT-associated markers included in the study. Among the plakins, desmoplakin (DSP), a key plakin with an established role in EMT [51,52], was included both at the protein and mRNA level. Among the EMT-associated proteins, established EMT markers such as TGFβ1 [53] and EGFR [54] were included. At the mRNA level, a broader range of EMT markers such as SNAI1/2, TWIST, and ZEB1/2 [53,54] was also included [55,56]. In general, a weak correlation was indicative of an r value between 0 and 0.3 (or −0.3), moderate between 0.3 and 0.5, and very strong above 0.5. The plakin-associated proteins catenin β1 (CTNNB1) [57], annexin A9 (ANXA9) [58], Rho, and protein kinase Cα (PRKCA) [59] were included alongside CA125 (MUC16) and GAPDH (GAPDH) [60,61].

The core plakin family members were compared to the classical and alternative markers of EMT at the protein (Figure 15) and mRNA levels (Figure 16). The plakins showed a range of correlations with the classical EMT markers. DSP had the strongest anti-EMT correlations at the protein level, being moderate-strongly associated with ECAD and strongly negatively associated with VIM (Figure 15). PPL and EVPL had weaker positive associations with ECAD. None of the plakins had a strong correlation with NCAD, but DSP showed a weak positive correlation with NCAD. Whereas DSP was strongly negatively associated with VIM, PLEC had an opposing moderately positive correlation. Regarding TGFβ1, weak to moderate positive correlations with PLEC and VIM were observed, but weak to moderate negative correlations with DSP and ECAD were observed. Weak to moderate positive correlations with EGFR were observed in all plakins except DSP (Figure 15).

Of the remaining proteins, GADPH showed a strong correlation with PPL expression, and MUC16 was consistently weak to moderately positive in its correlation with all plakins (Figure 15). GAPDH was included as a downstream target of p53 [61] involved in anti-plakin caspase activation and involved in the mTOR pathway, which has EMT implications [62]. EMT-associated CTNNB1 (β-catenin) has a strong positive correlation with DSP and ECAD and a moderate negative correlation with VIM and TGFβ1, consistent with its relationship with DSP. CTNNB1 expression as part of adheren junctions is key to the initiation of desmosome formation involving DSP. Whereas DSP can act as a tumour suppressor by inhibition of Wnt/β-catenin signalling [63]. The PPL-binding partner ANXA9 (member of the Ca^2+^-sensitive phospholipid-binding annexins) [64] showed a strong positive correlation with PPL in Type II EOC and moderate correlation with the other plakins. Whereas the signalling molecule PRKCα showed moderate positive association with PLEC but weak negative correlation with DSP (Figure 15). This suggests that plakin behaviour is nuanced within the group with differing levels of influence of EMT initiators and markers.

In terms of mRNA (Figure 16), only weak positive correlations were present between the plakin mRNA, EMT markers, and EMT-associated transcription factors (Figure 14), with PLEC being the highest of the plakin group. TGFβ1 mRNA had a moderate positive correlation with PLEC and EVPL (Figure 16). MUC16 and PRKCA both had moderate positive correlations with EVPL, and MUC16 also with PLEC. EVPL and PLEC also had the strongest positive correlation within the plakin mRNA expression.

### 3.6. Impact of Plakins and EMT-Associated Protein Expression on Survival of Patients

**Figure 17:**
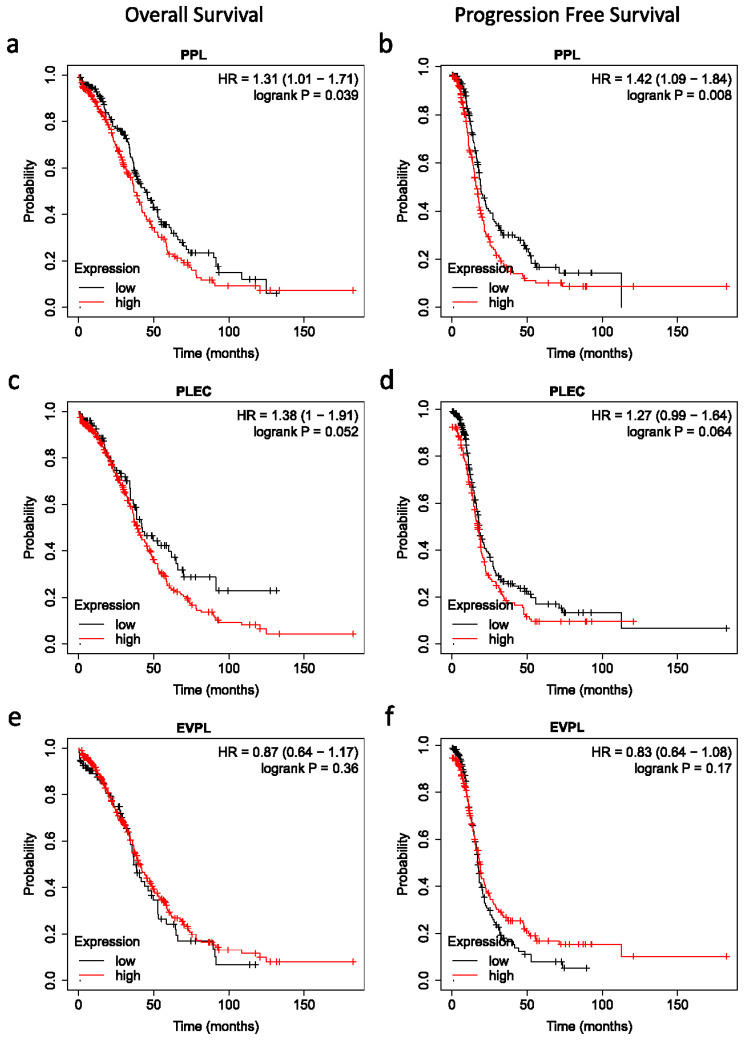
Kaplan–Meier analysis of overall and progression-free survival of patients in relation to mRNA expression of plakins. Cases analysed using the Kaplan–Meier Plotter website, dataset titled Ovarian Serous Cystadenocarcinoma (TCGA, Nature 2011) [49], with these conditions: WHO Type II serous ovarian cancer with p53 mutations, all treatment Types, and auto-threshold selection of high versus low expression. (**a**) PPL mRNA expression in relation to overall survival, (**b**) PPL mRNA expression in relation to progression-free survival, (**c**) PLEC mRNA expression in relation to overall survival, (**d**) PLEC mRNA expression in relation to progression-free survival, (**e**) EVPL mRNA expression in relation to overall survival, (**f**) EVPL mRNA expression in relation to progression-free survival. Sample numbers: n = 418 for OS, n = 398 for PFS, samples without relevant data were omitted.

**Figure 18:**
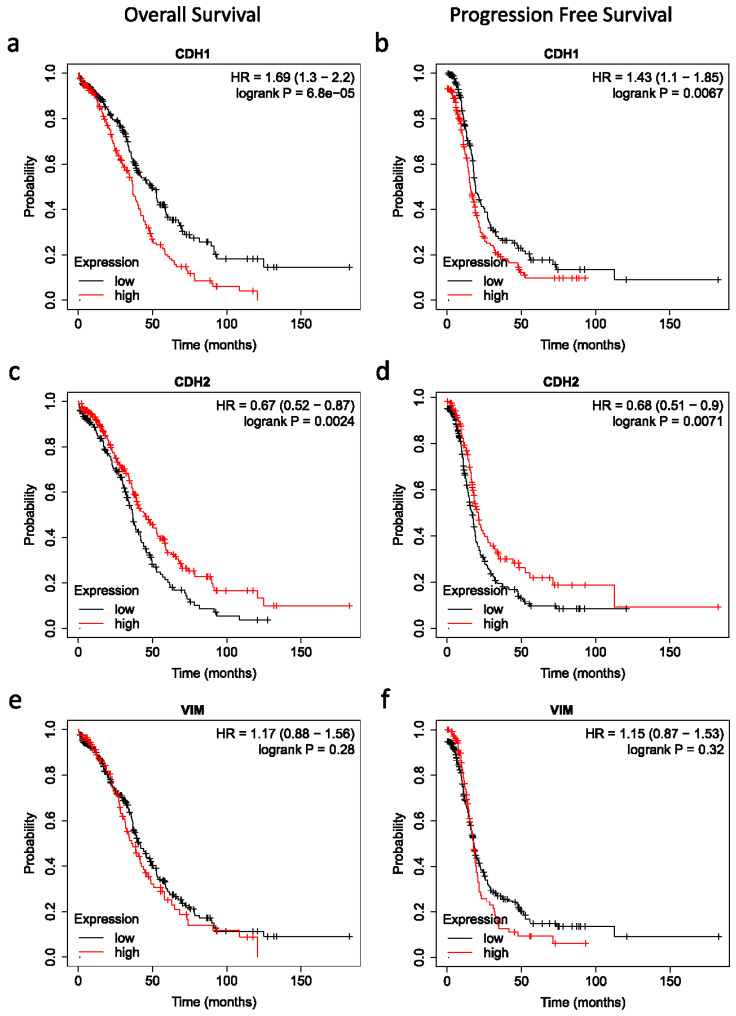
Kaplan–Meier analysis of overall and progression-free survival of patients in relation to mRNA expression of EMT markers. Cases analysed using the Kaplan–Meier Plotter website, dataset titled Ovarian Serous Cystadenocarcinoma (TCGA, Nature 2011) [49], with these conditions: WHO Type II serous ovarian cancer with p53 mutations, all treatment types, and auto-threshold selection of high versus low expression. (**a**) ECAD mRNA expression in relation to overall survival, (**b**) ECAD mRNA expression in relation to progression-free survival, (**c**) NCAD mRNA expression in relation to overall survival, (**d**) NCAD mRNA expression in relation to progression-free survival, (**e**) VIM mRNA expression in relation to overall survival, (**f**) VIM mRNA expression in relation to progression-free survival. Sample numbers: n = 418 for overall survival, n = 398 for progression-free survival, samples without relevant data were omitted.

Kaplan–Meier overall survival and progression-free survival based on the mRNA expression of PPL, EVPL, and PLEC, as well as ECAD, NCAD, and VIM were drawn from the TCGA database (Figure 17 and Figure 18). A high mRNA expression of PPL was associated with significantly low overall survival and progression-free survival in patients (Figure 17a,b), implying that low mRNA expression of PPL was beneficial for survival. On the other hand, no significant mRNA expression differences of EVPL and PLEC had any impact on overall and progression-free survival outcomes in patients (Figure 17c–f).

Surprisingly, high expression of NCAD had a significant beneficial impact on both overall and progression-free survival of patients (Figure 18c–d), implying that higher mRNA expression of NCAD was a good prognostic indicator for EOC patients. However, no such significant differences for ECAD and VIM could be noted for the EOC patients.

**Figure 19:**
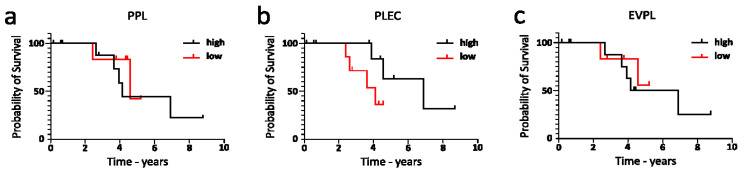
Overall survival in relation to high and low expression of PPL (**a**), PLEC (**b**), and EVPL (**c**) in RWH cases (WHO Type II only). Data analysis and survival graphs produced with GraphPad Prism 10.2.0 software of the protein expression and survival data of the WHO Type II cases presented in Figure 1, Figure 2, Figure 3, Figure 4, Figure 5, Figure 6 and Figure 7. Cases separated by level of plakin protein expression, high—above mean identified in Figure 2, low—below mean in Figure 3. No significant difference was found using Mantel–Cox (log-rank) test.

In Figure 19 the overall survival of our in-house cases was compared with the plakin protein expression level. Although there was insufficient data to establish statistical significance, a clear trend of beneficial high PLEC expression can be seen in Figure 19.

## 4. Discussion

The functional biology of cancer cells is dictated by the intracellular and external signals received by the cells and is very much dependent on cell–cell communication, which can ultimately modulate the course of cellular function. However, little is known about how this complex process modulates molecules such as plakins to install changes in cellular function such as EMT, which instigates the metastatic progression. In this study, we attempted to understand if the expression of plakins and their connections as adaptive receptors can assist in the downstream signalling changes that are required for the initiation of EMT in EOC. In short, we explored whether cell–cell communication in relation to plakin expression correlates with the process of EMT in EOC.

It is reasonable to expect that plakin expressions would be retained in solid tumours because of their roles in cell–cell and cell–ECM junctions [65]. In this study we demonstrate that the expression of PPL and EVPL is lower in the benign samples than in the Type I tumours. The benign human tissues are an approximate parallel to normal tissues, with similar ECM, adherent and cytoskeletal biology. It is suggested that in the benign samples, the cells are in a stable phase of growth with established cell junctions. Thus, their need for plakin production could be lower than the tumourigenic cells in Type I cancer that are more rapidly dividing. This difference is significant in PPL expression but also detectable in EVPL. In healthy tissues, PPL and EVPL are more highly expressed in stratified squamous epithelia than simple epithelia [65]. In our samples, the benign controls more closely matched simple epithelia and thus have less PPL and EVPL than multi-layered epithelial tumour cells. The most interesting results are the significant decrease in PPL and PLEC expression in Type II compared to Type I tumours. This may occur as tumour cells are more dispersed in Type II tumours and possibly are not connected so densely as in Type I tumours. Thus, there is an increased potential to break plakin–cell cytoskeletal organisation in a bid to disseminate from the origin of the tumour.

We demonstrate that there is a significant decrease in PPL expression between the Silverberg borderline and grade 3 tumours. Serous borderline tumours are intermediate tumours between benign and malignant tumour behaviour [66,67]. These tumours have molecular profiles very similar to Type I tumours and, if invasive, are classified as such. In summary, the less aggressive and invasive phenotypes of borderline tumours, coherent with Type I tumours, retain significantly higher levels of PPL expression compared to aggressive Type II and grade 3 tumours.

Similarly, tumours classed as surgical stage 1 have significantly higher PPL expression than those in higher FIGO stages of tumour dissemination, with a similar trend observed in the expression of PLEC. However, no significant difference in EVPL expression was observed. This loss of PLEC and PPL in Type II compared to Type I tumours suggests an increasing lack of cytoskeletal and structural stability, such as reduction of desmosome and hemidesmosome strength, within the Type II tumours. Additionally, there is potential for reduced cell–cell communication and number of cell junctions to enable sloughing of tumour cells for metastatic spread. The loss of PLEC expression may reduce its ECM connections with resultant increased dissemination of tumour cells, consistent with increasingly poor outcomes for overall survival.

However, the results described in our study are contrary to the results presented in a recent study where the mRNA and protein expression revealed significantly enhanced PPL expression in ovarian tumours compared to normal ovaries [68]. The discrepancy between that study and ours is the use of control normal ovaries in the previous study, which virtually lacked any obvious ovarian surface epithelium for both mRNA and protein analyses [69]. In contrast, by immunohistochemistry analysis, we demonstrate that PPL staining is confined to the epithelial cells of benign tumours as well as other ovarian tumours without any obvious staining in the surrounding tissues. Hence, analysis of the whole tissue in the case of normal ovaries, where ovarian surface epithelium constitutes a single layer, may not provide a justifiable comparison with ovarian tumours, in which epithelial tumours comprise a major portion of the tissue. In addition, the previous study also lacked a detailed demonstration of PPL expression in different stages, grades, and Types of ovarian cancer to portray the epithelial-cell confinement of PPL expression.

We demonstrate a significant positive correlation between PLEC and ECAD and a negative correlation between PLEC and NCAD in our investigation, with similar observations with PPL. We observed a trend of survival benefit with increased PLEC expression with our in-house Type II samples. Thus, strong PLEC expression corresponds to inhibited EMT changes in more aggressive EOC.

Reduced PLEC expression has been shown to inhibit keratin organisation and activate the Erk pathway [69]. Alongside KRT17, PLEC is associated with the integrin/FAK/Src/ERK/β-catenin axis [70] and may relocate to focal adhesions (FAs) and interact with VIM at FAs when disrupted from hemidesmosomes [71]. It is hypothesized that loss of PLEC and PPL in solid tumours would potentially increase the exposure of tumour cells to pro-EMT regulators (cytokines, chemokines, growth factors, etc.) in the TME to facilitate metastasis and tumour progression.

Plakin loss enables increased exposure to the influence of hypoxia, chemokine, and mechano-sensitive signals that instigate tumour cells onto the path of EMT and E-M plasticity due to reduced cell–ECM and cell–cell junctions, loss of anchoring stability implicating mechanosensitive signalling, and likely contributing to a looser tumour structure and subsequent exposure of the tumour cells to surrounding TME and the aberrant stimuli contributed by the evolving TME. As discussed previously, PLEC and PPL are associated with several cellular receptors and signalling pathways, including MAPK. Of note is PLEC involvement with CXCR4 activation [72], where CXCR4 is overexpressed in chemoresistant EOC [73] and associated with disease progression [74] and EMT initiation [75,76].

It may be postulated that an increase in tumour cell proliferation in advanced stages and grades of OC leads to immature cell–cell junctions and thus lower plakin expression. A consistent expression level of ECAD and NCAD was observed across both Type I and Type II tumours and in the metastatic omental deposits of Type II tumour cells. However, a difference was detected in NCAD expression when the cases were separated into FIGO surgical stages, suggesting a lower NCAD expression in tumours that had spread beyond the ovaries (stages 3–4). As our samples are primary solid tumours, this observation may reflect lower NCAD levels in tumour cells ‘left behind’ after dissemination within the peritoneal cavity. On the other hand, the expression of VIM varied markedly across the Type II tumour samples, but without significance in comparison to benign, Type I, and omental metastases. In metastatic tumour deposits in omentum, VIM protein expression appeared higher than in primary ovarian tumours, likely due to small tumour deposits and a high proportion of VIM-positive stromal cells and adipocytes. PPL protein expression was similar, but potentially decreasing, between ovarian and omental tumour cells. Change in plakin expression level does not seem to correlate with changes in the expression of conventional EMT markers, at least in the solid tumour samples included in our study.

On the contrary, the loss of PPL and PLEC expression may reduce the plakin–ECM connections with resultant increased dissemination of tumour cells. This postulation is consistent with the results showing that PLEC expression is significantly positively correlated with ECAD expression, and PPL expression shows a correlative trend with ECAD expression. Both PLEC and PPL protein expression are negatively correlated with NCAD expression. This is consistent with the retention of PLEC protein conferring a visible survival benefit. This may not only be through maintenance of cell stability but also the signalling role that PLEC plays in regulating cellular functions. However, loss of plakins may enable shedding of cells into the peritoneal cavity, enabling exposure of these cells to pro-EMT signals in EOC TME during tumour progression. Solid tumours with high levels of PLEC and PPL correlated significantly with low NCAD, suggesting loss of plakins may precede some of the EMT changes, and retention of plakins may inhibit or delay pro-EMT changes. This is consistent with work in colorectal cancer demonstrating that loss of PPL increases EMT potential, and PPL overexpression limits both tumour cell proliferation and EMT marker expression [42].

The ovarian cell lines used in this study originated from patients diagnosed with EOC, with OVCAR4 and OVCAR5 confirmed as high-grade/WHO Type II and derived from ascites. The two cell lines OVCAR4 and OVCAR5 are most similar in cell behaviour and plakin expression. They both form regular confluent monolayers with typical epithelial morphology of single-layer round cells, requiring contact with neighbouring cells for viability. The CAOV3 cell line is a considerable slow-growing cell line, which in monolayer culture grows as epithelial cobblestone-like clumps of 2–3 cells high. CAOV3 has overall moderate plakin levels, with potentially high PPL mRNA expression. This cell line also had the highest expression of ECAD amongst the tested cell lines. On the other hand, the HEY cell line is the most mesenchymal in appearance, has classical mesenchymal markers, high NCAD and VIM expression, and is consistently at the low end of plakin expression. This reflects its rapid ability to grow as elongated mesenchymal-like cells with lesser contact with the extracellular matrix, suggesting higher content of PPL-rich desmosomes are readily found in the more epithelial-like cell lines. This is consistent with our previous observation of high expression of PPL in epithelial cell-rich, low-grade/state/Type I tumours.

Our TCGA protein correlation analysis of plakins with EMT markers showed DSP as the most strongly linked plakin to an anti-EMT profile of positive ECAD, negative VIM, and weak positive NCAD correlation. A weak negative correlation between DSP and TGFβ1 protein, and effectively no correlation with EGFR, suggests that DSP may not be involved with TGFβ1 and EGFR-initiated pathways associated with EMT in EOC. DSP has been noted as tumour suppressor in lung cancer [63]. Consistent with that, DSP had the least correlation with EMT transcription factor mRNA (SNAI1/2, TWIST1, ZEB1/2). However, PLEC had higher but moderate positive correlation with the mRNA expression of these transcription factors. In contrast to DSP, PLEC protein had moderate to strong correlation with VIM, TGFβ1, and EGFR protein. On the other hand, PPL has a weak correlation with most of the EMT transcription factors described in the study at the mRNA level and a similar moderate correlation with EGFR protein as PLEC, but a weaker correlation with ECAD than DSP. As both PLEC and PPL can bind VIM directly, a greater correlation in expression was expected. Although not studied in our solid tumour tissue samples, DSP clearly has strong connections to both the maintenance of epithelial traits and the EMT process, being a target of ZEB53 and involved in the Wnt/β-catenin pathway [52]. As β-catenin is required to escort ECAD to the plasma membrane, the strong positive correlations seen between DSP, ECAD, and β-catenin support a role for DSP expression on the epithelial tendencies of EOC cells. It is plausible that the diminishing expression of PPL and PLEC with EOC progression may enable epithelial–mesenchymal plasticity of EOC cells in the EMT spectrum, but not the classical EMT process.

The observation of UALCAN CPTAC data in this study is in line with the complicated biology of OC progression, which is consistently in transition between the epithelial–mesenchymal and mesenchymal–epithelial states. Although it has been suggested and shown that primary EOC tumours undergo an EMT-like process during disease progression and retain a mesenchymal phenotype in advanced tumours [24], EOC cells in ascites retain epithelial features and are able to invade [77]. In that context, ECAD-expressing ovarian carcinoma spheroids have been shown to adhere to and invade the surrounding mesothelium [78]. However, a study using OC cell lines in vitro elegantly demonstrated that cell lines enriched in mesenchymal genes are competent in mesothelial clearance, while those enriched with epithelial signatures lack the capacity to do so [79]. Enhanced expression of transcription factors SNAI1, TWIST1, and ZEB1, which orchestrate the EMT process, promoted mesothelial clearance in cell lines with incompetence in mesothelial clearance, while knockdown of the EMT regulatory transcription factors TWIST1 and ZEB1 attenuated mesothelial clearance in OC cell lines with enhanced mesothelial clearance ability [79]. These observations suggest that mesenchymal features in EOC cell lines are a prerequisite for mesothelial clearance in a bid to start invasion. However, the paradigm of epithelial–mesenchymal plasticity exists in EOC tumours, which is reflected in this study and more so in patients’ overall survival and progression-free survival data obtained from TCGA, which showed low PPL mRNA and high NCAD mRNA expression to be good prognostic indicators for patients. However, the low mRNA levels in the TCGA data are suggestive of the mRNA levels in the high-grade/stages of tumours from different patients and do not relate to the comparative expression levels in relation to tumour stages/grades and Types in individual patients. Given the known role of plakins in EMT and the intricacy in detecting EMT-mediated processes in metastatic ovarian tumours, the expectation that plakin biology would play a crucial role in EOC progression still stands but remains to be elucidated.

## 5. Conclusions

In conclusion, this study found significantly lower expression levels of the plakins, PLEC and PPL, in Type II compared to Type I ovarian tumours. As the expression of these plakins is indicative of strong cell–cell and cell–ECM connections, this is consistent with the expression of plakins in Type I EOC, which has more structured architecture and more stable disease. The loss of PPL and PLEC in Type II tumours may result from the loss of cell–cell and cell–ECM connections during tumour progression and possibly a preparative mode for EOC cell dissemination to metastatic sites. There was no significant difference in expression of EMT markers between Type I and Type II EOCs. However, in Type II cases, high PLEC and often PPL expression were consistent with the retention of ECAD expression and low NCAD expression. PLEC and PPL both have cellular signalling roles in addition to consolidating desmosomes (PPL) or forming hemidesmosomes (PLEC), and we postulate that loss of PPL and PLEC may affect some of the plakin-associated signalling receptor-mediated effects that may influence cancerous changes in EOC cells during tumour progression. Our data suggest that changes in plakin expression and potentially the associated signalling pathways precede EMT changes in EOC cells.

## Figures and Tables

**Table 1:** Primers used for mRNA analysis.

Gene Name	Forward 5′3′ Primers	Reverse 5′-3′ Primers	Size
18S	GTA ACC CGT TGA ACC CCA TT	CCA TCC AAT CGG TAG TAG CG	153 bp
PLEC	TAC TAC CGC GAG AGT GCA GA	TCC TTG ATG GCG TTG ATG TA	212 bp
PPL	AGT GAC CTC CTT GGT GTC GT	AGG GTG AAT GAT GGT TGG G	153 bp
PPL	RT2 qPCR primer assay (200 tests)	Qiagen proprietary primer	138 bp
EVPL	RT2 qPCR primer assay (200 tests)	Qiagen proprietary primer	107 bp
Vimentin (VIM)	CCT ACA GGA AGC TGC TGG AA	GGT CAT CGT GAT GCT GAG AA	198 bp
E-Cadherin (ECAD)	AAA CAG CAA CGA CGG GTT AG	CTT AGG ATT GGG GGC AAA AT	81 bp
N-Cadherin (NCAD)	GGC ACA GAT GGT GTG ATT ACA G	GTC CCA GGC GTA GAC CAA GAA A	195 bp

## Data Availability

The datasets presented in the manuscript are part of a PhD thesis. If required, the data can be obtained from the corresponding author or Federation University, Australia, Mount Helen Campus library on request.

## Abbreviations

ECAD	E-cadherin
ECM	Extracellular matrix
EMT	Epithelial–mesenchymal transition
EOC	Epithelial ovarian cancer (excluding ovarian cancers from non-epithelial origins)
EVPL	Envoplakin
FIGO	International Federation of Gynaecology and Obstetrics
HGSOC	High-grade serous ovarian cancer (WHO type II)
LGSOC	Low-grade serous ovarian cancer (WHO type I)
MET	Mesenchymal–epithelial transition
NCAD	N-cadherin
OC	Ovarian cancer
OSE	Ovarian surface epithelium
PLEC	Plectin
PPL	Periplakin
TCGA	The Cancer Genome Atlas
TME	Tumour microenvironment
VIM	Vimentin
WHO	World Health Organisation
